# Comparative efficacy of two poeciliid fish in indoor cement tanks against chikungunya vector *Aedes aegypti *in villages in Karnataka, India

**DOI:** 10.1186/1471-2458-11-599

**Published:** 2011-07-28

**Authors:** Susanta K Ghosh, Preethi Chakaravarthy, Sandhya R Panch, Pushpalatha Krishnappa, Satyanarayan Tiwari, Vijay P Ojha, Manjushree R, Aditya P Dash

**Affiliations:** 1 National Institute of Malaria Research (ICMR), Poojanahalli, Kannamangala Post, Devanahalli, Bangalore-562110, Karnataka, India; 2 Bangalore Medical College and Research Institute, Bangalore-560002, Karnataka, India; 3 Office of the District Health and Family Welfare Services, District Tumkur 572101, Karnataka, India; 4 World Health Organization, South East Asia Regional Office, Indra Prastha Marg, New Delhi-110002, India

## Abstract

**Background:**

In 2006, severe outbreaks of *Aedes aegypti*-transmitted chikungunya occurred in villages in Karnataka, South India. We evaluated the effectiveness of combined information, education and communication (IEC) campaigns using two potential poeciliid larvivorous fish guppy (*Poecilia reticulata*) and mosquitofish (*Gambusia affinis*), in indoor cement tanks for *Aedes *larval control.

**Methods:**

Trials were conducted in two villages (Domatmari and Srinivaspura) in Tumkur District from March to May 2006 for *Poecilia *and one village (Balmanda) in Kolar District from July to October 2006 for *Gambusia*. A survey on knowledge, attitude and practice (KAP) on chikungunya was initially conducted and IEC campaigns were performed before and after fish release in Domatmari (IEC alone, followed by IEC + *Poecilia*) and Balmanda (IEC + *Gambusia*). In Srinivaspura, IEC was not conducted. Larval surveys were conducted at the baseline followed by one-week and one-month post-intervention periods. The impact of fish on *Aedes *larvae and disease was assessed based on baseline and post-intervention observations.

**Results:**

Only 18% of respondents knew of the role of mosquitoes in fever outbreaks, while almost all (*n *= 50 each) gained new knowledge from the IEC campaigns. In Domatmari, IEC alone was not effective (OR 0.54; *p *= 0.067). Indoor cement tanks were the most preferred *Ae. aegypti *breeding habitat (86.9%), and had a significant impact on *Aedes *breeding (Breteau Index) in all villages in the one-week period (*p *< 0.001). In the one-month period, the impact was most sustained in Domatmari (OR 1.58, *p *< 0.001) then Srinivaspura (OR 0.45, *p *= 0.063) and Balmanda (OR 0.51, *p *= 0.067). After fish introductions, chikungunya cases were reduced by 99.87% in Domatmari, 65.48% in Srinivaspura and 68.51% in Balmanda.

**Conclusions:**

*Poecilia *exhibited greater survival rates than *Gambusia *(86.04 *vs*.16.03%) in cement tanks. Neither IEC nor *Poecilia *alone was effective against *Aedes *(*p *> 0.05). We conclude that *Poecilia *+ IEC is an effective intervention strategy. The operational cost was 0.50 (US$ 0.011, 1 US$= 47) per capita per application. Proper water storage practices, focused IEC with *Poecilia *introductions and vector sanitation involving the local administration and community, is suggested as the best strategy for *Aedes *control.

## Background

In 2006, severe outbreaks of chikungunya occurred in the villages in Karnataka, South India. Chikungunya is a rare arboviral infection transmitted by *Aedes *mosquitoes. It shares the same vector and geographical distribution as dengue fever, and has similar symptoms with the exception of incapacitated arthralgia [1]. Over a period of three decades, chikungunya outbreaks have struck the southwestern islands of the Indian Ocean region. In India, outbreaks occurred late-2005 mainly in the southwestern states and were primarily transmitted by *Aedes aegypti *[2]. Approximately1.4 million suspected cases were reported in 2006 and over 0.7 million cases were from Karnataka alone [3]. Larvivorous fish in natural habitats that feed on *Anopheles *larvae have been successfully used in malaria control [4], but their use in confined domestic containers against *Aedes *larvae has been very limited [5]. Based on our previous experience using two potential poeciliid larvivorous fish guppy (*Poecilia reticulata*) and mosquitofish (*Gambusia affinis*) for malaria control in Karnataka [4,6], we aimed to conduct two small-scale community-based feasibility studies combined with information, education and communication (IEC) to test the comparative efficacy of these fish in containing *Ae. aegypti *larval infestation and reducing chikungunya. Use of *Poecilia *fish combined with IEC offered the most effective means of controlling *Aedes *mosquito populations in our study.

## Methods

In brief, our studies were carried out within Karnataka in two villages (Domatmari and Srinivaspura) in Tumkur District and in one village (Balmanda) in Kolar District, affected by chikungunya. A survey on knowledge, attitude and practice (KAP) on chikungunya was initially conducted in Domatmari and Srinivaspura. IEC campaigns were performed in Domatmari and Balmanda, before and after fish introductions. *Aedes *larval surveys were carried out in all three villages to assess the impact of the intervention measures undertaken. *Poecilia *was released in Domatmari and Srinivaspura, and *Gambusia *in Balmanda. Fish survival was also monitored during the larval surveys. Anonymised data on chikungunya cases were collected from the respective district health offices.

### Study areas and the population

Studies were conducted in two highly chikungunya-affected districts, Tumkur and Kolar, between March and October 2006. Domatmari (population 2040; 420 households) and Srinivaspura (population 568; 114 households), under the Venkatapura (population 74,680) Primary Health Centre (PHC) in Pavagada taluka^1 ^(14°6'LN 77°16' LE, average elevation of 750 masl) of Tumkur, and Balmanda (population 1342; 284 households), under the Kamasamudram (population 36,484) PHC in Bangarpet taluka (12°58' LN 78°12' LE, average elevation of 850 masl) of Kolar, were randomly selected. The study areas are dry, rocky, sandy reddish brown soils and drought prone, with low irregular rainfall (600-800 mm) during 60 to 72 rainy days per annum. Temperature ranges from 13 to 39°C. Most households studied were comprised of three to six individuals, with a male to female sex ratio of 1:0.97. Agriculture is the main form of employment for the local villagers. Literacy rate varies between 58 and 72%.

### Tumkur trial

#### KAP survey

In March 2006, a KAP survey on a representative sample of 50 respondents (mean age 28 ± 15, range 12-68; 31 males and 19 females) was conducted in Domatmari and Srinivaspura of Tumkur District. Unprompted and pre-tested questionnaires were used for this survey.

#### IEC campaigns

In Domatmari, an IEC campaign on chikungunya, its treatment and management, mode of transmission and control of *Aedes *larvae, especially using larvivorous fish and water storage practices, was organized to determine its impact on vector abundance. A health education campaign was performed using lectures and a live demonstration of larvivorous fish feeding on mosquito larvae. Live larvae collected from households were also shown to the villagers, and maintenance of the fish within small water-storing tanks was further explained. Inter-personal communication with each household was established during each monitoring survey. Impact of the IEC campaign was assessed after one month of the trial on 50 respondents (mean age 23 ± 11, range 11-62; 28 males and 22 females). In Srinivaspura, no IEC activity was organised for comparison.

### Kolar trial

Chikungunya outbreaks also occurred in the neighbouring Kolar District in May-June 2006. Within this district, Balmanda was one of the chikungunya-affected villages randomly selected for the *Gambusia *trial from July to October 2006. IEC campaigns were performed as described

above for Domatmari. IEC impact was assessed based on 50 respondents (mean age 24 ± 12, range 11-59; 33 males and 17 females) at the end of the trial. *Gambusia *was introduced after the IEC campaign.

#### *Aedes *larval surveys

At the beginning of the study, *Aedes *larval surveys were conducted in every fourth line-listed house at the baseline (before IEC and fish introduction) and one-week post-IEC campaigns. Repeat larval surveys were also conducted one-week and one-month after fish introduction. Larvae were collected using a white enamel bowl (300 ml) and a torch light. A one-larva-per-container method was used for our larval survey and density per-dip was further calculated. Finally, the impact of fish introduction on the *Aedes *larval infestation was estimated based on house index (HI), container index (CI) and Breteau index (BI) [2].

#### Fish release and monitoring

*Poecilia *were collected from a stream near Ventakapura, whereas *Gambusia *were collected from a pond in Kamasamudram that was already in use in the malaria control programme [6]. For each of the selected villages, 10-15 fish were released into each indoor cement tank. Both fish species were used for all households (*n *= 818). *Poecilia *were released in 482 tanks in Domatmari and 32 in Srinivaspura, whereas *Gambusia *in 337 tanks in Balmanda. Monitoring of fish survival was simultaneously conducted with larval surveys.

#### Fish impact on disease

Under the National Vector Borne Disease Control Programme, anonymised data on chikungunya cases were collected from the respective district health offices. Each office gave permission to use this data.

#### Ethical approval

The Institutional Ethics Committee of National Institute of Malaria Research, New Delhi provided ethical approval for the KAP survey. Community consent was obtained for the entire study, and especially fish introductions, from the local village leaders and *Gram Panchayat *(village-elected council) members, school teachers and a religious leader.

#### Statistical analysis

Frequency of breeding contribution for each habitat and the impact of fish on larval density, and responses of the IEC campaigns, were analysed using Fisher's Exact and χ^2 ^tests. Larval density data were not normally distributed and were consequently subjected to a non-parametric Kruskal-Wallis test. Significance values (*p*) for percentage differences (baseline and post-intervention) were computed using on-line Java Script tests on difference paired proportion estimates from a set of random paired observations for the intervention parameters, i.e. HI, CI and BI (http://home.ubalt.edu/ntsbarsh/Business-stat/otherapplets/PairedProp.htm). Results with a *p *< 0.05 were considered to be statistically significant.

## Results

### KAP survey and impact of IEC campaigns

KAP survey results (Table 1) revealed that only18% respondents understood the role of mosquitoes during fever outbreaks, and only 2% responded that controlling mosquitoes could contain these outbreaks. Two-thirds of the respondents believed that mosquitoes breed in stagnant dirty water. Almost all go to the hospital when they get fever and believed that the administered medicine would cure them. Two-thirds of households stored water in indoor cement tanks, which were cleaned less than once a week, and 78% of the respondents covered the water storage containers. Over half of the population protected themselves from mosquito bites using coils, fans or burning of Neem (*Azadirachta indica*) leaves. A few respondents (8%) knew the role of larvivorous fish in mosquito control.

**Table 1:** Knowledge, Attitude and Perception (KAP) on chikungunya in villages in Tumkur District, Karnataka, India, March 2006

Questionnaire	Respondents (*n *= 50)
1. Do you know about the fever in your area?	Yes-39 (78.0%) No-11 (22.0%)
2. Do you know what causes this fever?	No-36 (72.0%) Dengue fever -3 (6.0%) Dirty air -1 (2.0%) Rats-1 (2.0%) Mosquitoes-9 (18.0%)
3. Do you know that mosquitoes spread this fever?	No-39 (78.0%) May be- 2 (4.0%) Yes-9 (18.0%)
4. What do you do when someone in your family gets this fever?	Go to hospital-48 (96.0%) Not certain- 2 (4.0%)
5. What do you think is the cure for this fever?	Control mosquitoes-1 (2.0%) Medicine-46 (92.0%) Drinking water- 1 (2.0%) Do not know-2 (4.0%)
6. Do you know where these mosquitoes breed?	Stagnant dirty water-33 (66.0%) Cracks in walls-1 (2.0%) Earth and air -1 (2.0%) Do not know- 15 (30.0%)
7. What are the water storage facilities in your house?	Cement tanks-33 (66.0%) Metal/Plastic containers-9 (18.0%) Earthen pots- 7 (14.0%) Overhead tanks-1 (2.0%)
8. Do you cover them?	Yes-39 (78.0%) Sometimes-2 (4.0%) No-9 (18.0%)
9. How often do you clean and dry the containers?	Less than 1 week-33 (66.0%) More than 1 week -14 (28.0%) Non-response-3 (6.0%)
10. How do you protect yourself from mosquito bites in day and night?	No protection-24 (48.0%) Protection*-26 (52.0%)
11. Do you know that fish can control mosquito larvae?	Yes - 4 (8%) No - 46 (92%)
12. Will you co-operate if we introduce control measures?	Yes-50 (100.0%)

*Protection measures-Coils, nets, fans, Neem (*Azadirachta indica*) smokes.

The impact of IEC on fish introductions is summarized in Table 2. Almost all people studied gained newly acquired knowledge after the IEC campaigns in Domatmari and Balmanda villages (*p *> 0.001). People also gained knowledge that the current outbreaks were due to chikungunya transmitted by the *Aedes *mosquito, which breeds in clean water (*p *> 0.01). The *Poecilia *programme in Domatmari was more liked than the *Gambusia *programme in Balmanda (*p *< 0.001). The unpleasant smell caused by dead *Gambusia *fish in the water storage containers in Balmanda was the main draw back suggested for this programme (*p *< 0.001).

**Table 2:** Responses of post-IEC after fish introductions in villages in Karnataka, India, March to October 2006

Questionnaire	Response		
	
	Domatmari (*n *= 50)	Balmanda (*n *= 50)	*p value*
1. Have you gained new knowledge through IEC?	Yes - 48 (96%) No - 2 (4%)	Yes - 47 (94%) No - 3 (6%)	> 0.001
2. Did you know the cause of the fever outbreak?	a. Chikungunya - 42 (84%) b. Dengue - 8% (16%) c. Malaria - 0 d. Brain fever (JE) - 0	a. Chikungunya - 39 (78%) b. Dengue - 3 (6%) c. Malaria - 4 (8%) d. Brain fever (JE) - 2 (4%)	a. > 0.001 b. < 0.01 c. < 0.01 d. < 0.01
3. Which mosquito species was responsible for the fever?	a. *Anopheles *- 3 (6%) b. *Aedes *- 47 (94%) c. *Culex *- 0	a. *Anopheles *- 6 (12%) b. *Aedes *- 42 (84%) c. *Culex *- 2 (4%)	a. < 0.01 b. > 0.001 c. < 0.01
4. *Aedes *mosquitoes breed in	a. Clean water - 46 (92%) b. Polluted water - 4 (8%)	a. Clean water - 39 (78%) b. Polluted water - 11 (22%)	a. > 0.01 b. < 0.01
5 Did you like the fish-based biocontrol programme?	a. Yes - 44 (88%) b. No - 6 (12%)	a. Yes - 26 (52%) b. No - 24 (48%)	a. < 0.001 b. < 0.001
6. What are the drawbacks of such programme?	a. Bad smell - 4 (8%) b. Fish died - 2 (4%)	a. Bad smell - 34 (72%) b. Fish died - 31 (62%)	a. < 0.001 b. < 0.001
7. Would you promote such programme?	a. Yes - 50 (100%) b. No - 0	a. Yes - 32 (64%) b. No - 18 (36%)	a. < 0.01 b. < 0.001

### *Aedes *larval baseline survey

Initially, a baseline survey was carried out in all villages to determine the most potential breeding habitats for *Aedes *mosquitoes (Table 3). A total of 456 containers in 231 households were surveyed in all of the studied villages. Percentage breeding contribution indicated indoor cement tanks supported maximal larval breeding [86.9%, relative risk (RR) 8.2, χ^2 ^6.74, *p *< 0.001] followed by earthen pots (16.8%, RR 6.7, χ^2 ^8.23, *p *< 0.001), outdoor cement tanks (8.0%, RR 3.2, χ^2 ^8.67, *p *< 0.001), and metal and plastic containers (3.8%, RR 4.4, χ^2 ^7.38, *p *< 0.001). Larval emergence data indicated 99% of the larvae were *Ae. aegypti *and 1% was *Ae. albopictus*.

**Table 3:** Baseline *Aedes *larval surveys in villages in Karnataka, India, March and July 2006

Type of breeding habitats	% Breeding contribution	RR value	χ^2 ^(df)	*p *value
Indoor Cement tank	86.9	8.2	6.74 (3)	< 0.001
Earthen pots	16.8	6.7	8.32 (3)	< 0.001
Outdoor Cement tank	8	3.2	8.76 (3)	< 0.001
Metal and plastic containers	3.8	4.4	7.38 (3)	< 0.001

RR: Relative risk, df: degree of freedom.

### Survival of fish

Data on fish survival are summarized in Table 4. In all villages studied, a nearly 100% fish survival rate was observed after one week of fish release. However, the rate varied in the respective villages in the one-month post-fish release. In Domatmari, the rate was 86.04% (95% CI: 78.1-94.6%, *p *= 0.27), 33.7% in Srinivaspura (95% CI: 23.7-42.3%, *p *< 0.001) for *Poecilia *and only 16.03% (95% CI: 11.4-23.8%, *p *< 0.001) in Balmanda for *Gambusia*.

**Table 4:** Sustainability of larvivorous fish in villages in Karnataka, India, March to October 2006

Village	Number of tanks released fish	Number of tanks checked/fish present (%)		
		
		After one week	After one month	*p *value
Domatmari	482	178/176 (98.8) [95% CI 96.3-99.4)	172/148 (86.04) [95% CI 78.1-94.6]	0.27
Srinivaspura	32	26/25 (96.1) [95% CI 94.3-98.4]	27/8 (33.7) [95% CI 23.7-42.3]	< 0.001
Balmanda	337	126/124 (98.4) [95% CI 96.1-99.2]	131/21 (16.03) [95% CI 11.4-23.8]	< 0.001

CI: Confidence Interval.

### Impact on *Aedes *larval populations

Mean larval densities of *Aedes *(per dip) at the baseline in Domatmari, Srinivaspura and Balmanda were 9.2 (95% CI: 7.4-12.2), 10.6 (95% CI: 7.8-14.4) and 14.3 (95% CI: 11.2-16.4), respectively. In all villages, no larvae were detected one-week post-fish release. After one month of intervention the mean larval densities were 0.2 (95% CI: 0.08-0.4, *p *> 0.001) in Domatmari, 7.8 (95% CI: 4.8-9.7, *p *< 0.05) in Srinivaspura and 11.7 (95% CI: 8.5-14.1, *p *< 0.05) in Balmanda.

### Impact on *Aedes *larval breeding

**Figure 1:**
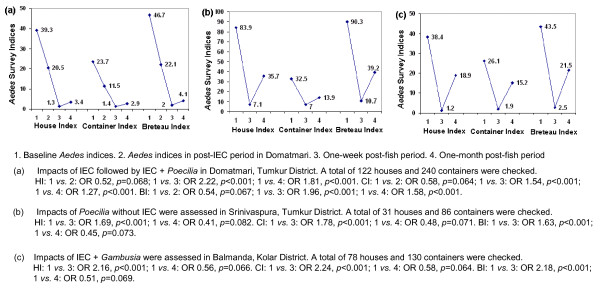
**Impact of poeciliid larvivorous fish against *Aedes aegypti *larvae in indoor cement tanks in villages in Karnataka, India, March to October 2006**.

The impact of larvivorous fish on *Aedes *larvae is summarized in Figure 1. In Domatmari, 240 containers within122 houses were checked during the baseline survey. Similarly, 86 containers within 31 houses were checked in Srinivaspura, and 130 containers within 78 houses were checked in Balmanda. Considering BI as an important parameter, IEC alone did not effectively control larval breeding in indoor cement tanks in Domatmari [odds ratio (OR): 0.54, *p *= 0.067]. Very significant evidence of improved larval control was observed in the one-week and one-month post-*Poecilia *release + IEC periods (OR: 1.96 and 1.58, *p *< 0.001). In Srinivaspura (*Poecilia *alone), no such improvement was observed one-month post-*Poecilia *release (OR: 0.45, *p *= 0.073) after a significant improvement in the one-week period (OR: 1.63, *p *< 0.001). In Balmanda, *Gambusia *+ IEC proved effective up to the one-week period (OR: 2.18, *p *< 0.001), but did not sustain larval control one-month post-fish release resulting in additional *Aedes *breeding (OR: 0.51, *p *= 0.069).

### Impact on the disease

In the baseline survey, a total of 816 fever cases showing chikungunya symptoms were recorded in Domatmari, while 226 cases were identified in Srinivaspura and 432 cases in Balmanda. After one month of intervention only one case (reduction 99.87%) in Domatmari, 78 cases (65.48%) in Srinivaspura, and 136 cases (68.51%) in Balmanda were recorded.

## Discussion

Before the study began, more than 3000 cases of fever were reported in villages within the Tumkur and Kolar Districts. Subsequent laboratory investigations confirmed these outbreaks were caused by chikungunya [2]. Thus, the present study was devised to identify an alternative method of vector control, as no other immediate options were available. Accordingly, we decided to use larvivorous fish as a biocontrol method against *Aedes *larvae as this was found very effective against malaria vectors [4,6]. While we ensured matched control villages were included in our study as per standard experimental protocol, during the course of the trial, participants within the control villages released fish into their tanks when they discovered that the fish were responsible for controlling on-going outbreaks in the study villages. Thus, after consulting a statistician, the impact of intervention was assessed considering the data before and after fish introductions.

Self-sustained populations of *Poecilia *and *Gambusia *are the most preferred poeciliid larvivorous fish in malaria control in India [4,6]. Besides their predatory nature, the presence of such fish may also inhibit *Ae. aegypti *oviposition in domestic containers [7]. Maintaining these fish in confined habitats is important in an *Aedes *control programme. In the one-week post-fish release period, an almost 100% fish survival rate was recorded in all villages. In the following one-month period, fish populations were better sustained in Domatmari than in Srinivaspura and Balmanda.

*Poecilia *is an omnivorous species that better survives in confined habitats, namely open dug wells. In the absence of larvae, this fish can survive on other food available within the ecosystem. It also grows equally well in small containers with minimum care. In the malaria control programme we observed that villagers offered cooked rice or Ragi (a type of millet locally grown) as a food supplement, which helped to sustain and propagate their fish populations. This information was further told to the villagers as part of the IEC programme. In contrast, *Gambusia *is a cannibalistic species that feeds on zooplankton as its preferred food source, and populations of this fish are not sustained in small water bodies for long periods of time [8]. This was possibly one of the reasons that this fish did not survive even for a month in cement tanks. Thus, *Gambusia *is not a preferred fish in small water habitats, and is preferable for large water bodies such as ponds and lakes [6]. While *Poecilia *reproduced in some of the study tanks, indicating sufficient conditions for sustained populations in these containers, this was not observed for *Gambusia*. We have also observed this finding in the laboratory (unpublished observation).

Many biocontrol agents have been tested against *Aedes *larvae. Recent experience in Vietnam has been remarkably successful, with members of the community being closely engaged in vector control efforts by cleaning public areas and using biocontrol agents in water storage tanks [9]. There is an abundance of local *Mesocyclops *spp. (copepods) in Vietnam that can be incorporated into specifically designed community-based control programmes aided by *Micronecta *water bugs and fish [10]. In a village in French Polynesia, *Mesocyclops aspericornis *and poeciliid fish released in ponds and tanks successfully controlled mosquito species other than *Aedes*, which do not breed in these habitats [11]. In a coastal village in Taiwan, integrated control of *Aedes *used *G. affinis, P. reticulata, Tilapia mossambica *and *Sarotherodon niloticus *in potable water containers. They were later replaced with *Cyprinus carassius *because of constant availability and adaptability [12]. In 1980, Chinese catfish was used to control *Ae. aegypti *larval breeding when a dengue outbreak occurred in fishing villages among Chinese coastal provinces [13]. In Thailand, the most effective method of *Ae. aegypti *control was maintaining fish in rectangular tanks and correctly covering water storing containers with lids [14]. In Southern Mexico, five indigenous fish species, namely *Lepisosteus tropicus, Astyanax fasciatus, Brycon guatemalensis, Ictalurus meridionalis *and *P. reticulata*, were significantly effective as biocontrol agents against *Ae. aegypti *larvae in water storage tanks [5]. More recently, the Dengue Control Programme in the northeastern Brazilian state of Ceará has used five non-native larvivorous fish species (*Betta splendens, Trichogaster trichopteros, Astyanax fasciatus, P. sphenops*, and *P. reticulata*) to combat *Ae. aegypti *larval infestation [15]. Another example was reported in Cambodian villages, where *P. reticulata *reduced dengue-carrying *Ae. aegypti *larval infestation by 79%, compared to control villages [16].

### Water tanks and water storing practices

**Figure 2:**
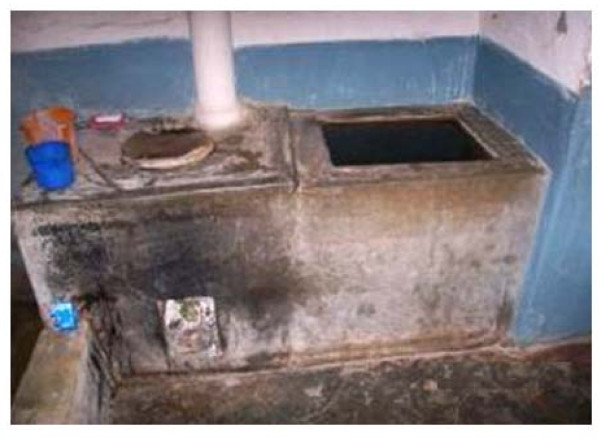
**An example of an indoor cement tank attached to an oven for heating water**. Water temperature in such tanks is maintained at 24-26°C, which is favourable for *Aedes *larval breeding.

**Figure 3:**
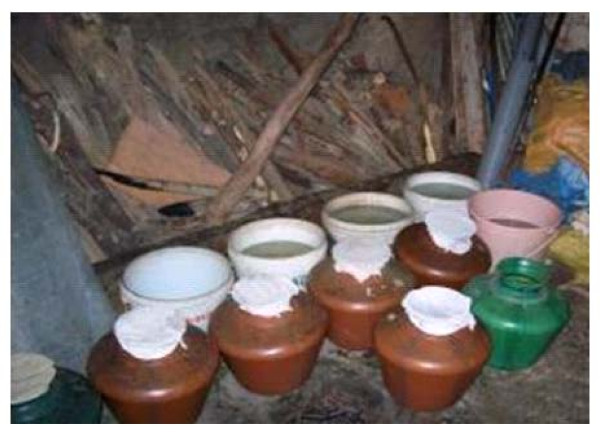
**Villagers covered their water containers after the IEC campaign, thereby preventing *Aedes *from larval breeding**.

In each village, water is supplied via a deep bore well system operated by the local *Gram Panchayats*. Due to irregular water supplies, the villagers store water in indoor cement tanks for washing, bathing and also for drinking. *Aedes *mosquitoes mainly breed within these tanks. On an average 2.6 ± 0.6 (range 0-6) indoor cement tanks were recorded in each household. Two-thirds of villagers cleaned their tanks once in a week (as per the KAP survey), and IEC alone did not reduce the level of *Aedes *larval breeding. This is because of a faulty design in these tanks, in which the base of each tank is lower than the ground level making it difficult to completely empty. A few inches of water is retained at the bottom of the tanks, allowing *Aedes *larvae to rest at the bottom during the cleaning of mosquito-positive tanks. Moreover, each tank is attached to an oven for heating water for bathing (Figure 2). This helps maintain a favourable temperature range of 24-26°C, which is conducive for the growth and development of mosquito larvae, especially *Aedes*. Other breeding habitats, namely outdoor cement tanks that were placed under shades for cattle feeding, small plastic containers, and earthen pots where fish could not be introduced, also supported *Aedes *larval breeding. This knowledge was conveyed to villagers through IEC campaign in an effort to prevent larval breeding (Figure 3).

**Figure 4:**
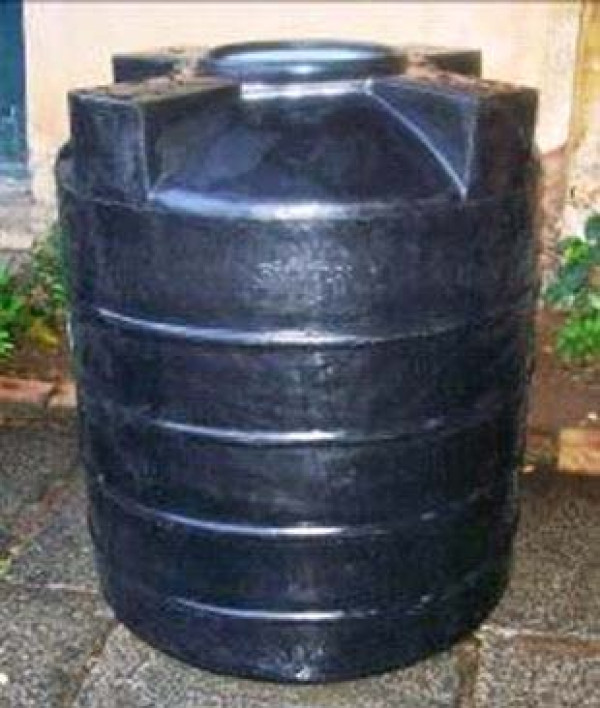
**Commercially available plastic moulded mosquito-proof water storage tank**.

Currently, commercially available plastic moulded mosquito-proof water storage tanks may be used for the prevention of mosquito breeding (Figure 4). One-month observations provided by our study suggested that *Poecilia *introductions in water storage tanks combined with IEC is an alternative method of *Aedes *control. IEC improved the survival and handling of this fish at the community level. This was not observed when using *Gambusia. Poecilia *are available in many villages in Karnataka used in the malaria control programme, and can be easily grown in wells and small tanks. Furthermore, the local religious trust propagated *Poecilia *in their garden tanks and helped supply them to the local villagers. After completion of the trials, *Poecilia *was reintroduced after IEC in Srinivaspura and *Gambusia *was replaced with *Poecilia *in Balmanda for control of *Aedes *larvae.

During the control programme, a weekly application of an organophosphorus compound (Temephos; 1 ppm per litre) is recommended for the control of *Aedes *larvae in potable water. Being a chemical insecticide, this is not liked by many and is logistically difficult to use including its prohibitive costs. Moreover, *Aedes *can potentially develop resistance to this compound if used for a long period, as reported in Brazil after 30 years of its use [17]. Larvivorous fish are therefore suggested as the best option for controlling *Aedes *larval infestation, as they are both sustainable and cost effective. The operational cost was calculated at 0.50 (US$ 0.011, 1 US$= 47) per capita per application. Thus, monthly monitoring and application of *Poecilia *may be recommended after proper IEC. Consequently, the community is likely to facilitate the long-term control of mosquitoes, thereby preventing the diseases transmitted by these vectors.

## Conclusions

Our study successfully determined the comparative efficacy of artificially maintaining populations of two non-native fish to control mosquito vectors. The use of *Poecilia *+ IEC is further highly recommended for areas still affected by chikungunya in Karnataka [18], while *Gambusia *should be avoided as populations of this species are not sustained in domestic containers. Effective *Aedes *control should be formulated on the basis of local situations. Therefore, day-to-day water storing practices, 'vector sanitation' involving the National Rural Health Mission, the *Gram Panchayat*, and local community are essential for a successful and sustainable programme.

## Endnote

^1 ^taluka is a secondary revenue division of a district.

## Competing interests

The authors declare that they have no competing interests.

## Authors' contributions

SKG, ST and VPO conceived and arranged the entire programme. PC, SP and PK conducted the KAP survey and monitoring. SKG, ST, VPO and MR carried out entomological studies and fish release. APD reviewed and edited the paper. SKG assisted in data analysis. All authors helped write, read, and approved the final manuscript.

## Pre-publication history

The pre-publication history for this paper can be accessed here:

http://www.biomedcentral.com/1471-2458/11/599/prepub
